# Modern Electrophysiological Methods for Brain-Computer Interfaces

**DOI:** 10.1155/2007/56986

**Published:** 2007-11-25

**Authors:** Rolando Grave de Peralta Menendez, Quentin Noirhomme, Febo Cincotti, Donatella Mattia, Fabio Aloise, Sara González Andino

**Affiliations:** ^1^Electrical Neuroimaging Group, Department of Clinical Neurosciences, Geneva University Hospital, 1211 Geneva, Switzerland; ^2^Neurodynamics Laboratory, Department of Psychiatry and Clinical Psychobiology, University of Barcelona, 08035 Barcelona, Catalonia, Spain; ^3^Neurofisiopatologia Clinica, Fondazione Santa Lucia, 00179 Roma, Italy

## Abstract

Modern electrophysiological studies in animals show that the spectrum of neural oscillations encoding relevant information is broader than previously thought and that many diverse areas are engaged for very simple tasks. However, EEG-based brain-computer interfaces (BCI) still employ as control modality relatively slow brain rhythms or features derived from preselected frequencies and scalp locations. Here, we describe the strategy and the algorithms we have developed for the analysis of electrophysiological data and demonstrate their capacity to lead to faster accurate decisions based on linear classifiers. To illustrate this strategy, we analyzed two typical BCI tasks. (1) Mu-rhythm control of a cursor movement by a paraplegic patient. For this data, we show that although the patient received extensive training in mu-rhythm control, valuable information about movement imagination is present on the untrained high-frequency rhythms. This is the first demonstration of the importance of high-frequency rhythms in imagined limb movements. (2) Self-paced finger tapping task in three healthy subjects including the data set used in the BCI-2003 competition. We show that by selecting electrodes and frequency ranges based on their discriminative power, the classification rates can be systematically improved with respect to results published thus far.

## 1. INTRODUCTION

Development of direct brain-computer interfaces (BCI) is a novel and very interesting field of research, aimed at building an alternative communication channel between men and machines that do not rely on physiological output pathways. EEG-based BCI stems from decades of research on electrophysiological correlates of brain activity. As such, it is mostly based on methods developed for traditional analysis of scalp signals. Such techniques resulted were beneficial in the earliest states of EEG-based BCI and speeded up initial developments. However, when compared with the accelerated progresses achieved with implanted devices, a certain impasse becomes evident [1]. Few attempts have been made to incorporate results obtained in electrophysiological studies in animals within this field. For instance, EEG-based BCI is mostly characterized by the use of regions (i.e., electrodes locations) and frequency bands defined from average evoked potentials (e.g., P300), and thus mainly determined by the EEG low-frequency components [2]. Such a priori preselection of a few electrodes based on results of ERPs is at odds with recent experimental findings showing substantial learning induced modification of neural activity within a single session [3, 4] or the involvement of a distributed brain network in even the simplest motor tasks.

EEG analysis aiming to answer basic neurophysiological questions has benefited from analysis procedures that rely upon the broad spectrum analysis of single trials either based on scalp-recorded signals or noninvasive estimates of local field potentials (eLFPs) [5–7]. Such procedures offer an alternative to traditional electrophysiological analysis using trials averaging and/or single maps (or set of maps) analysis able to unveil new neural mechanisms. The application of these methods to paradigms used for standard ERP analysis demonstrated that short EEG windows (500 milliseconds) following a single stimulus presentation are enough to identify the category of the visual stimuli and the brain regions involved in their processing [8]. Even shorter time windows (200 milliseconds) were sufficient to predict the laterality of the impending hand responses in simple visuomotor reaction-time tasks [9]. Later study demonstrated the importance of neural oscillations above 100 Hz for the decoding of movement laterality. Parallel studies based on invasive recordings in animals and humans confirmed the importance of such largely unexplored frequency band in movement control [10, 11]. In practice, the use of features derived from the broad band spectral analysis of the signals reduces the importance of the preselected pattern recognition algorithm allowing for implementations based on simpler and faster classifiers.

Previously described procedures have been incorporated in a Matlab-based environment for the analysis of both online (near real-time) and offline EEG data. This platform, dubbed as Geneva Brain-Computer Interface (GBCI), contains the novel methods proposed for the analysis of single trials, based on broad spectrum exploration of the EEG or ELFPs derived from it, that is, based on sound electrophysiological and biophysical information together with simple and fast classification algorithms.

In this paper, we illustrate the application of these principles to three different data sets. The first data set consisted in EEG recordings from a paraplegic patient suffering from complete traumatic spinal cord injury. In the experiment, the patient attempts to control the movement of a cursor on the screen through imagined movements of the limbs and self control of the mu-rhythm. The second and the third data sets correspond to EEG data recorded from healthy subjects performing a self-paced finger tapping task. The second data set (two subjects) was recorded at our lab and the third data set is a courtesy of Blankertz and colleagues [12, 13]. Latest data set constitutes a good reference to evaluate the advantages of the proposed procedures since it has been analyzed by many different groups on the framework of the BCI competition 2003 [13].

## 2. MATERIAL AND METHODS

### 2.1. Data recording

The first data set (referred from now on as MI) was acquired from a subject (male, 42 years old, suffering from paraplegia due to complete traumatic spinal cord injury at level of T10. EEG signals were collected by 61 sintered-silver electrodes mounted on a cap according to the extended 10–20 system, amplified and digitized at 1000 samples per second (BrainAmp, Brain Products GmbH, Gilching, Germany). The subject seated relaxed at his own wheelchair while performing the experiment. The task consisted in moving a cursor towards a target as soon as the latter appeared on any of the four sides of the screen. The instructions received at the beginning of the training in order to move the cursor were to concentrate on kinesthetic imagination of movement of his hands (cursor up), his feet (cursor down), his right or left hand (cursor right or left, resp.). At the time of recording, the subject had automated control of his mu-rhythm, thus he reported that he only sporadically had to imagine movements. Nevertheless, during acquisition, the experimenter made sure that no overt limb movement was present and that EEG potentials were not contaminated by EMG or EOG. In addition, EEG recordings were reviewed offline by an expert electroencephalographer to remove epochs contaminated by EOG and EMG artifacts. EMG and EOG control was based on the monitoring of all border (e.g., T7, O1) and frontopolar channels, respectively.

Only correct trials of the (two) classes composed by right and left cursor movements linked to lateralization of the mu-rhythm were analyzed. The data set was acquired in four sessions recorded in different days, and consisted of about 240 trials (120 for each class, resp.). Each trial, starting at target appearance, ended when the cursor either hit the target (correct) or moved to the wrong side of the screen (incorrect). Trial duration ranged between 2 to 10 seconds. We here restrict the analysis to the one second window starting one second after target appearance.

The second data set (referred from now on as ENG) corresponds to two different subjects (ENG1 and ENG2) performing a self-paced finger tapping paradigm identical to the one used for the BCI-2003 competition (see below). EEG was recorded from 64 electrodes disposed in standard (10/10) system using a sampling frequency of 512 Hz. A total of 240 epochs (120 for each hand) of 500 milliseconds were selected for analysis per subject. To avoid EMG contamination, epochs ended 146 milliseconds before key press.

The third EEG data set (referred from now on as BCI) is the self-paced finger task of the BCI-2003 competition [12]. It was recorded from a healthy subject seated in a normal chair, relaxed arms resting on the table, and fingers in the standard typing position at the computer keyboard. The task was to press keys with either the index or the little finger of either the left or the right hand in self-chosen order and timing. A total of 416 epochs of 500 milliseconds were selected for analysis. To avoid EMG contamination, epochs ended 130 milliseconds before key press. The training set was composed by 316 epochs randomly selected and the remaining 100 epochs were used as the test set. Twenty eight electrodes disposed in standard (10/20) system were used for the EEG recording at 1000 Hz.

### 2.2. Feature extraction

Practical experience on EEG-based BCI indicates that subjects can learn to control specific frequency rhythms as to provide control of neuroprosthetic devices [14–17]. Nonetheless, electrophysiological recordings in animals show that oscillatory activity at frequency bands hardly explored on human EEG encode relevant neurophysiological information [18]. Indeed, very high-frequency oscillation above 100 Hz, sometimes called epsilon oscillations [19], correlate with motor intentions [9]. We therefore use as feasible physiological features all frequency oscillations identified from the power spectral density (PSD) of the EEG. To select from the whole PSD the range of oscillations and sensors that better encode the mental commands specific to each task, we use a mathematical algorithm (the Discriminative Power) described below (see Section 3.3).

We computed the PSD using modern multitaper methods [20]. These methods have shown to be particularly well suited for spectral analysis of short segments of noisy data, and have been successfully applied to the analysis of neuronal recordings in behaving animals [21]. Specifically, the PSD was estimated using 7 Slepian data tapers to reduce the variance. Each EEG window was multiplied by eachof the tapers, and the Fourier components were then computed via Fast Fourier Transform (FFT). The power spectral density was then computed by taking square of the modulus of the FFT from 0 to Nyquist frequency (i.e., half of the frequency sampling).

### 2.3. Feature selection

Features were selected on the basis of their Discriminative power (DP) [19]. This measure provides an estimate of the percentage of true positives that can be obtained classifying with each single feature given that the number of false positive is set to zero. By definition, the DP does not consider interaction between features and might be affected by extreme values (outliers). However, in practice, these outliers are very unlikely. If outliers are indeed present, they can be identified and removed by simple exploration of the training or learning set.

To compute the DP, we denote by a (b) the feature vector for class A (B), that is, a vector formed by the feature values over all trials in class A (B). By swapping vectors *a* and *b*, we can always assume that $a_{\min}$ = {minimum of *a*} ≤ $b_{\min}$ = {minimum of *b*}. If $b_{\max}$ ={maximum of *b*} ≤ $a_{\max}$ = {maximum of *a*} (i.e., one condition contains the other), then DP = 0; otherwise,

(1) $$\text{DP}=\frac{\text{card}\{a<b_{\text{min}}\,\}+\text{card}\{b>a_{\text{max}}\,\}}{\text{card}\{a\}+\text{card}\{b\}}*100,$$

where card {⋅} stands for the number of elements in a set.

**Figure 1:**
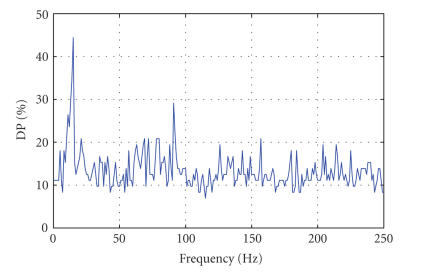
Discriminative power (DP) versus frequency for the MI data set (paraplegic patient). The panel represents the plot of maximum DP (best discrimination between left and right cursor movements) as a function of frequency. Although the trained mu-rhythm provides the best discrimination in this patient, significant contribution to the discrimination (higher than 20% of trials) is observed for very fast frequency oscillation (peaks at 69, 72, 78–80, 91, and 157 Hz).

**Figure 2:**
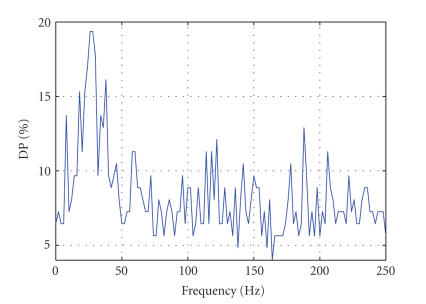
Discriminative power (DP) versus frequency for the ENG1 data set. The panel represents the plot of maximum DP (best discrimination between left and right finger tappings) as a function of frequency. Peaks (DP *>* 12) are seen at alpha, beta, and gamma bands but also for very high-frequency bands (122 and 188 Hz).

**Figure 3:**
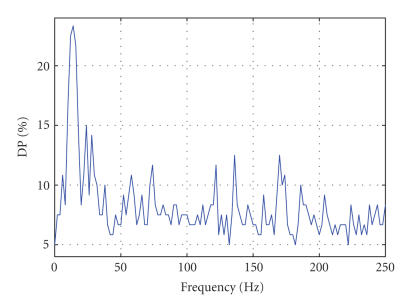
Discriminative power (DP) versus frequency for the ENG2 data set. The panel represents the plot of maximum DP (best discrimination between left and right finger tappings) as a function of frequency. Peaks (DP *>* 12) are observed at classical frequency bands (alpha and beta) as well as epsilon oscillations (136 Hz and 170 Hz).

**Figure 4:**
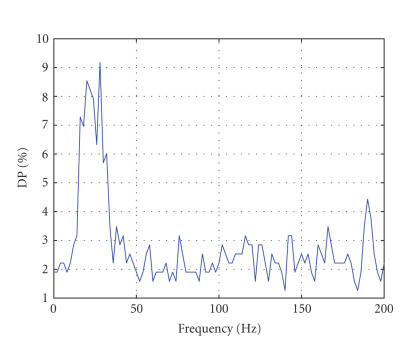
Discriminative power (DP) versus frequency for the BCI data set (BCI 2003). The panel represents the plot of maximum DP (best discrimination between left and right finger tapping) as a function of frequency. Discrimination is maximal over the beta/low gamma band with little discrimination for the ultra-fast frequency oscillations.

Given the matrix composed by the DP for all sensors and frequencies, we define the set of the best N features as the highest N entries of this matrix. Plotting the maximum DP for each column (i.e., all sensors confounded) as a function of the column index (frequency) yields a very informative plot summarizing the behavior of each frequency over the whole electrode space (see Figures 1, 2, 3, and 4).

### 2.4. Support vector machine classifiers

For the sake of simplicity and speed, we used a linear classifier: the linear proximal support vector machine (PSVM) developed and implemented in [22]. As described by Mangasarian and Wild [23], “ … a standard support vector machine with a linear classifier, is given by a plane midway between two parallel bounding planes that bound two disjoint half spaces each containing points mostly of class 1 or 2. In another somewhat less standard approach, the proximal support vector classification, two parallel planes are generated such that each plane is closest to one of two data sets to be classified and such that the two planes are as far apart as possible. The classifying plane is again midway between the parallel proximal planes.” Since these planes are determined by the unconstrained minimization of a quadratic function, the PSVM formulation leads to a very fast and efficient algorithm.

### 2.5. Crossvalidation procedure

The performance was evaluated with a 10-fold crossvalidation method where the data set is divided into ten subsets. Each subset is used once as test set while the complementary nine subsets are used as training sets to select the features and compute the classifier. Consequently, every data point (i.e., trial) is a member of the test set only once and a member of the training set nine times.

The correct classification (CC in %) rates reported here indicate the percentage of trials on the test set correctly assigned to its original class by the classifier. Unless otherwise specified, it corresponds to the CC value averaged over the 10 folds.

## 3. RESULTS

### 3.1. Results for MI data: mu-rhythm lateralization

Our goal in the analysis of this data set was to explore the possible role of high-frequency rhythms in a task where the subject has received training on mu-rhythm control. We considered two different strategies of analysis. First, to evaluate the generalization of the model independent of the recording session, we pooled the data from the four experimental sessions into one single data set.

Figure 1 shows a typical distribution of the maximum (over electrodes) DP values observed as a function of frequency on the training set. Significant contribution is observed all over the frequency axis with main peaks (higher than 20%) at 11–15, 69, 72, 78–80, 91, and 157 Hz.

The proportion of correct classification (CC%) for each fold was 75, 79, 83, 87, 87, 91, 83, 75, 83, and 75 with an average CC value of 82%. The differences observed between folds (from 75 to 91) suggest that the features and the classifier of a randomly selected part of the data might not be good enough to describe the full variability of the underlying process. Changes on the internal state of the subject such as motivation, attention, or switches on strategy between sessions might explain such results.

To further evaluate this aspect, we carried out a second analysis where each session was submitted separately to a 10-fold crossvalidation. The 10-fold averaged CC% results for the four sessions were 73, 86, 81, and 73, suggesting that the strategy used is not equally efficient for all sessions or that in addition the data is not homogeneous from session to session. Nevertheless, comparison with previous analysis suggests that a global model with features derived from all sessions together is more efficient than separate models for each session. More importantly, the DP plot in Figure 1 shows the importance of high frequencies for differentiating between conditions and should be then considered on any model of this data.

### 3.2. Results for ENG data: self-paced finger tapping

Through the analysis of this data set, we would like to illustrate the strategy and methods described before that rely on the single assumption that the EEG oscillatory activity contains the information needed to correctly classify the single trials into one of the two classes. Following the precept that scalp locations and frequencies should be selected on the basis of their capability to discriminate between classes, we computed for each electrode and each single trial the PSD as described in Section 2.2 and applied the 10-fold crossvalidation procedure described in Section 2.5

Figures 2 and 3 show typical distributions of the maximum (over electrodes) DP as a function of frequency for subjects ENG1 and ENG2, respectively. These are the results obtained over the training set. Significant contribution is observed at low (<50 Hz) and very high (>150 Hz) frequencies.

A discussion about the best approaches to select the optimal number of features is out of the scope of this paper that basically aims to stress the importance of minimizing assumptions when exploring the encoding value of EEG oscillatory activity. Thus, for the sake of simplicity, and just as a matter of example, we present in Table 1 the crossvalidation result (CC%) for some predefined number of features for both subjects.

By selecting the number of features from this table, we can obtain classification values comparable with or better than most (13 out of 15) results submitted to the BCI-2003 competition [13] for a similar task. A direct comparison using the data set included in the competition is presented in next section.

### 3.3. Results for BCI data: self-paced finger tapping

Based on the definition given by the organizers of the competition, we used the training set to compute the DP for all frequencies and all electrodes. The maximum DP over the electrodes as a function of the frequency is depicted in Figure 4. Surprisingly, the discriminative power maxima were observed for frequencies below 40 Hz although the frequency sampling of the data set was 1000 Hz. The differences with subjects ENG1 and ENG2 (see Figures 2 and 3) in terms of both frequencies and DP values are striking. As obvious from the plot, the features selected with the DP measure belong to the low frequencies 0–40 Hz for this subject. The DP selected the higher entries from the DP matrix composed by the 28 electrodes (rows) and the 40 frequencies (columns). To select the number of features, we explored the classification results on the training set as a function of the number of features.

**Figure 5:**
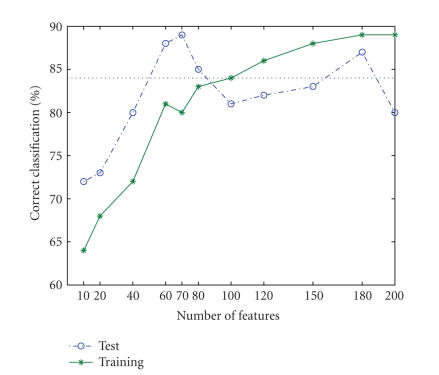
Selecting the number of features for the BCI data set. The picture depicts the percentage (%) of correct classification (CC) on the training set (continuous trace) and the test set (discontinuous trace) as a function of the number of features. The number of features (180) is defined as the beginning of the first plateau, that is, where increasing the number of features does not increase CC on the training set.

Figure 5 depicts the percentage (%) of correct classification (CC) on the training set and the test set as a function of the number of features. To be compatible with the information available at the time of the competition, we selected the number of features based only on the training set. For the number of features [10, 20, 40, 60, 70, 80, 100, 120, 150, 180, 200], we obtained CC values of [64, 68, 72, 81, 80, 83, 84, 86, 88, 89, 89], respectively. The final number of features was selected as 180, corresponding to the value where the CC first stabilizes (reaches a plateau) at a value of 89%. Note that, as happens with linear interpolation procedures, the CC might still increase with the number of features and attain a new plateau for a higher number of features. Nonetheless, for this number of features, the CC is 87% for the test set outperforming the best results obtained thus far for this data (i.e., best results are marked as a horizontal dotted line in Figure 5). The plot of the CC for the test set indicates that there are better solutions using only 60 or 70 features. At these points, performance on the test set attains 88% and 89%, respectively.

## 4. DISCUSSION AND CONCLUSIONS

The most impressive results obtained thus far in the brain control of neuroprosthetic devices have been probably those based on highly invasive recordings of action potentials within the motor cortex of monkeys [24]. In this study, Chapin et al. [24] demonstrated accurate control of a robotic arm through the decoding of the information contained on the spike trains. However, the tradeoff invasiveness/benefits of invasive approaches remains to be evaluated in practice. At this stage, noninvasive control modalities might offer a safer and cheaper alternative for patients. Of substantial interest for the community of researchers dealing with noninvasive BCI such as the EEG is the finding that very high-frequency oscillations are significantly correlated to the tuning of simultaneously recorded single units. This would imply that while synaptic activity will mainly contribute to the EEG lower frequencies [25], the EEG epsilon oscillations might contain significant power from action potentials. This finding is not exclusive to the motor cortex but seems to hold true for the inferotemporal cortex as well [26]. This finding is highly promissory since recent theoretical [27] as well as experimental studies [9, 28, 29] provide evidences that such very high-frequency oscillations are readily observable by scalp EEG recordings. This would imply that scalp EEG might constitute a more accurate BCI control modality than previously thought. To take practical advantage of these developments, we have to develop the analysis techniques that are able to separate such weak signals from the background noise and the algorithms required to readily interpret the mental commands from these signals. Importantly, very fast rhythms develop over short temporal windows and appear to constitute a crucial element for ultra-fast synaptic transmission [30]. Later finding would imply that the analysis windows required to detect low frequency (e.g., ≤ 40 Hz) cortical potentials might be replaced by shorter temporal windows needed for high frequencies (e.g., 150 Hz). This could increase the efficiency of noninvasive BCI systems.

In this paper, we have described how simple analysis techniques can be exploited to face the new challenges introduced by the need to analyze such broad band signals to extract their more informative features on individual basis. We used the multitaper spectral method as a way to provide more robust spectral estimates. This was combined with the Discriminative power measure which is enormously simple and still provides substantial information about the rhythms that better differentiate between the studied classes. Importantly, such feature selection alternatives can be entered into a linear classifier to fulfill the requirements of real-time control of neuroprosthetic devices.

The analysis of the finger tapping task data from the two subjects (ENG data) confirms the presence and the importance of high frequencies on the EEG. While the higher discrimination is observed for relatively low-frequency ranges (<50 Hz), the presence of peaks for gamma and epsilon (>100 Hz) frequency ranges is systematic. We hypothesize that it is the complementary character of the high and low frequencies that allow for good classification results using so simple procedures (i.e., linear classifiers). Nevertheless, further analyses are needed to confirm this conjecture.

On the light of the experimental results described above, the DP results obtained for the BCI-2003 finger tapping task are slightly surprising. Rhythms providing the best differentiation between tapping hands were limited to frequencies below 40 Hz. This is in clear contradiction with evidence from direct intracortical recordings in epileptic patients which indicate that the encoding of different motor actions might involve rhythms up to 180 Hz [31]. Similar conclusions have been obtained in monkey studies that demonstrate significant cosine tuning of very fast oscillations in both 2D and 3D center-out reaching tasks [10]. The DP plots of Figure 4 are also at odds with the results shown on Figures 2 and 3. In general, the DP values observed in this subject were lower than the ones we obtained for the ENG data and with a remarkably flat DP profile, that is, the DP was very similar for all frequencies with DP differences rarely surpassing 2%.

Despite the lack of oscillatory activity over 40 Hz, we obtained for this data set a correct classification rate of 87%. This rate constitutes a slight improvement in performance when compared with rates previously achieved for the same data set. The improvement is however noticeable if one considers the simplicity of the procedures employed for feature selection and the fact that results are based on very simple and fast (linear) classifiers.

The BCI data discussed here has been analyzed by several authors. The best rate obtained in the competition was 84% [13]. Posterior attempts [32] failed to improve these results despite combining source localization algorithms and more complex tools than the ones employed here. One possible explanation is the use of cumbersome preprocessing algorithms (spatial filtering, region of interests, etc.) aiming to substitute the classifier. Note that such preprocessing steps are likely to imply a heavier computational load than the simple linear classifier used here. A second aspect likely to influence their results is the selection of an inverse solution which is extremely sensitive to noise. According to our previous experience with inverse solutions, a sound regularization strategy is required to achieve good classification results on short single trials analysis windows [8, 33]. Nevertheless, we have observed that for the case of simple tasks like finger tapping or error-related negativity, resorting to inverse solutions is not needed since the scalp EEG contains all the information required for categorization of the single trials. The use of an inverse solution adds little information while introducing unnecessary computational load. This is probably why the classification rates in this study go beyond the results of Congedo et al. [32].

In the case of the paraplegic patients, we observed better classification accuracy when features were selected from the pooling of sessions. However, classification rates were not considerably reduced when features and classifier were computed for each session separately. While the most discriminative oscillations were observed within the mu-band that the subject has been trained to control, substantial discrimination was observed for the fast (gamma) and ultra-fast (above 100 Hz) rhythms. Considering the purported relationship observed between such oscillations and action potentials, we could interpret this result as an evidence of sustained action potential activity in the presence of imagined limb movements. In any case, these results show that training a given rhythm does not suppress the importance of self-generated oscillatory activity for the performance of imagined movements. To our knowledge, this is the first evidence of modulation of ultra-fast rhythms during imagined limb movements in a paraplegic patient.

This study illustrates that EEG-based BCI systems might considerably benefit from the experience gathered from animal electrophysiology. Rather than increasing the computational burden, broad band spectral analysis and individualized feature selection facilitate the use of simpler feature selection algorithms and linear classifiers. The observed modulation of ultra-fast frequency of oscillations in the paraplegic patient paves the way for studies aiming to clarify the functional role of these rhythms. If the relationship between single-unit activity and ultra-fast oscillations is confirmed, we might be able to provide a faster and finer control of neuroprosthetic devices in the future using noninvasive modalities.

## Figures and Tables

**Table 1:** 

Number of features	CC% for ENG1	CC% for ENG2
50	74	79
80	78	81
100	79	80
150	77	82
